# Versatile Zirconium Oxide (ZrO_2_) Sol-Gel Development for the Micro-Structuring of Various Substrates (Nature and Shape) by Optical and Nano-Imprint Lithography

**DOI:** 10.3390/ma15165596

**Published:** 2022-08-15

**Authors:** Nicolas Crespo-Monteiro, Arnaud Valour, Victor Vallejo-Otero, Marie Traynar, Stéphanie Reynaud, Emilie Gamet, Yves Jourlin

**Affiliations:** Université de Lyon, Université Jean-Monnet-Saint Etienne, Laboratoire Hubert Curien, UMR CNRS 5516, 42000 Saint-Etienne, France

**Keywords:** zirconium oxide, sol-gel, optical lithography, nano-imprint lithography, non-planar substrates, plastic

## Abstract

Zirconium oxide (ZrO_2_) is a well-studied and promising material due to its remarkable chemical and physical properties. It is used, for example, in coatings for corrosion protection layer, wear and oxidation, in optical applications (mirror, filters), for decorative components, for anti-counterfeiting solutions and for medical applications. ZrO_2_ can be obtained as a thin film using different deposition methods such as physical vapor deposition (PVD) or chemical vapor deposition (CVD). These techniques are mastered but they do not allow easy micro-nanostructuring of these coatings due to the intrinsic properties (high melting point, mechanical and chemical resistance). An alternative approach described in this paper is the sol-gel method, which allows direct micro-nanostructuring of the ZrO_2_ layers without physical or chemical etching processes, using optical or nano-imprint lithography. In this paper, the authors present a complete and suitable ZrO_2_ sol-gel method allowing to achieve complex micro-nanostructures by optical or nano-imprint lithography on substrates of different nature and shape (especially non-planar and foil-based substrates). The synthesis of the ZrO_2_ sol-gel is presented as well as the micro-nanostructuring process by masking, colloidal lithography and nano-imprint lithography on glass and plastic substrates as well as on plane and curved substrates.

## 1. Introduction

Zirconium oxide (ZrO_2_) is an intensively studied and used material due to its many remarkable physical and chemical properties. It has a high melting point (2700 °C), high hardness (between 11 and 18 GPa depending on the phase) [1,2], good chemical resistance [2,3], biocompatibility [4], high refractive index (2.1 at 633 nm) [5], wide band gap (5 eV) [6], high transparency in the visible and near-infrared range [7] and photoluminescence properties [8,9]. Due to its numerous properties, zirconium oxide is used in many applications such as protective coatings against corrosion, wear and oxidation [10,11], in optical applications (mirror, filters, etc.) [12,13,14,15], in anti-counterfeiting solutions [9] and in health applications such as the dental field [16,17].

There are many methods to synthesize ZrO_2_, among which we can mention reactive sputtering [18,19,20], chemical vapor deposition [21,22] and atomic layer deposition [23,24,25]. These techniques are well known in thin film deposition processes and are widely described in the literature. However, they do not allow micro- and nano-structuring in a simple way (without etching step) of these coatings to obtain complex patterns (according to the shapes, micro-nanostructures, etc.), which limits their use as well as their properties. Another process to elaborate ZrO_2_ thin films is the sol-gel method [26,27,28,29,30,31]. The sol-gel method has the advantage of micro-nanostructuring the films by optical lithography [29,31] or by nano-imprint lithography [32,33]. The optical lithography presents the advantage of being able to micro-nanostructure variable substrates of various shapes [29,30] and sizes [34,35] without having to resort to etching processes. Among the lithography processes, the masking lithography consists in obtaining a pattern by exposing it through a mask, letting the light pass through in the opened areas. This allows to structure quickly patterns on planar and non-planar substrates. The colloidal lithography consists in using silica nano-spheres to create photonic nano-jets underneath the layer of nano-spheres. These photonic nano-jets allow a field enhancement of the incident field, allowing the thin film micro-structuration according to the hexagonal and periodic pattern whose period is equal to the sphere diameter. If the material behaves like a positive resist, we achieve micro-holes [36] and if it behaves like a negative photoresist, we obtain nano-pillars [35,37]. Another method is nano-imprint lithography (NIL), which consists of patterning a layer by pressing a stamp (mould) on it [33]. This method has the advantage of being very fast, low cost and being able to adapt to a great number of supports and patterns. Nevertheless, it requires a sol-gel solution which can be patterned and stabilized by a thermal or UV treatment.

In this paper, we demonstrate how our ZrO_2_ sol-gel can be used to obtain both complex patterns (shapes, micro-nanostructures, etc.) by optical lithography (mask lithography, colloidal lithography) and by nano-imprint lithography. We also show the possibility of using this versatile sol-gel and the associated structuring methods to structure complex patterns on variable substrates in their nature and geometry.

## 2. Materials and Methods

### 2.1. Sol-Gel

To elaborate photo-patternable films, a mixture of two sols with different reactivities has been prepared. The first sol (sol 1) presents low chemical reactivity due to chelation of the alkoxide groups by BzAc. The second sol (sol 2) was prepared according to the procedure described thereafter, which yielded to sols very stable over time when stored, but very reactive when used for the fabrication of metallic oxide thin films. Sol 1 was prepared by reacting Zirconium (IV) propoxide (Zr(OPr)_4_ from Fluka) with 1-Benzoylacetone (BzAc from Aldrich) in anhydrous ethyl alcohol (EtOH from Aldrich). The Zr(OPr)_4_/BzAc/EtOH molar composition was 1/0.9/20. Sol 2 was obtained by mixing Zr(OPr)_4_ with deionized water, hydro-chloric acid (HCl from Roth), and butyl alcohol (BuOH from Merck) as a solvent. The Zr(OPr)_4_ concentration in the solution was 0.4 M, and the Zr(OPr)_4_/H_2_O/HCl/BuOH molar composition was 1/0.8/0.13/24. The sol was aged at room temperature for 2 days before being used. Finally, the photo-sensitive solution was prepared by mixing sols 1 and 2 to obtain a final sol with a Zr(OPr)_4_ concentration of 0.6 M and a BzAc/Zr(OPr)_4_ molar ratio of 0.6.

Then, the sol was deposited by using the spin-coating technique at a speed of 3000 rpm for 60 s, before being heated at 110 °C for 90 min, resulting in a so-called xerogel film, i.e., an inorganic polymer film made of Zr-O-Zr chains with organic chain-end groups arising from the sol formulation, mainly Zr-BzAc complexed species. The xerogel films are soluble in alcohol as far as BzAc stays complexed with Zr(OPr)_4_. The main interest of this protocol relies on the properties of BzAc, which makes the film soluble in a solvent while being sensitive to UVA light. Indeed, under UVA illumination, the Zr-BzAc complex is partially degraded into insoluble species. Therefore, it will create a contrast of solubility, widely described in the literature [38], between illuminated and non-illuminated areas when it is selectively exposed to UVA light, allowing us to easily structure our films at different scales.

### 2.2. Optical Lithography

#### 2.2.1. Macroscopic Mask Lithography

The pattern-based masks are macroscopic patterns made from a binary black-and-white image printed on a transparent plastic sheet using an inkjet printer, allowing various macroscopic patterns. In the first step, a ZrO_2_ xerogel layer was deposited by spin coating at 3000 rpm for 1 min. The mask was positioned above the xerogel layer during exposure to UV light. The whole area was irradiated using a UV lamp at a wavelength of 365 nm for 5 min at 200 mW/cm^2^. Only the transparent areas of the mask let the UV light pass through, thereby allowing the pattern to be transferred onto the xerogel film after development

#### 2.2.2. Colloidal Lithography

Silica microspheres of 1 µm diameter (in ethanol suspension (96% *v/v*) micro-mod) functionalized with a hydrophobic acrylate surface were deposited on ZrO_2_ xerogel thin films covered with a poly(methyl methacrylate) (PMMA) layer according to the Langmuir Blodgett (LB) approach [39]. To achieve this monolayer of silica microspheres deposited on the xerogel film, an LB machine (KSV NIMA LB) (Biolin Scientific) was used. The silica microsphere monolayer was spread on the aqueous sub-phase at room temperature and left for 10 min in order to let the solvent evaporate. After compression of the silica microsphere monolayer at a barrier speed of 3 mm/min using the LB machine, the microspheres were deposited on the thin film at a surface pressure of 40 mN·m^−1^ using the dipping method with a withdrawal speed of 3 mm/min. The ZrO_2_ xerogel layer was deposited by spin coating at 3000 rpm for 1 min and the PMMA layer was deposited by spin coating at 6000 rpm for 1 min. The PMMA layer was used to protect the ZrO_2_ thin film from water and to allow UV to pass through during film exposure. After deposition, the microspheres were illuminated at a wavelength of 365 nm for 90 s at 100 mW/cm^2^ in order to obtain ZrO_2_ nano-pillars with a 2D hexagonal arrangement [35].

### 2.3. Nano-Imprint Lithography

Polydimethylsiloxane (PDMS) stamps with sinusoidal micro-nanostructures of 800 nm period and 60 nm deep are used to micro-nanostructure the ZrO_2_ xerogel films. After deposition of the ZrO_2_ xerogel films by spin-coating at 4000 rpm for 30 s, PDMS stamp was applied to the ZrO_2_ xerogel films in a humidity- and temperature-controlled environment (20 °C and 50% humidity) under 1 bar of pressure for 3 min. Afterwards, a UV illumination at a wavelength of 365 nm for 5 min at 200 mW/cm^2^ is used to stabilize the patterned ZrO_2_ films.

### 2.4. Characterizations

The film structure was analyzed using Raman micro-spectroscopy (LabRam ARAMIS from Horiba Jobin Yvon company, Kyoto, Japan) with an excitation wavelength at 633 nm (He-Ne laser) and with a He-Cd laser (325 nm). The micro-nanostructured thin films were characterized by atomic force microscopy (AFM) measurements (Dimension Icon from Bruker company, Billerica, MA, USA)) in tapping mode with a tip AppNano (ACTA) and by scanning electron microscopy (SEM) using low vacuum mode coupled and LVSED detector with a JSM-IT80 from JEOL. The film thicknesses were measured by a profilometer Veeco Dektak 3 ST.

## 3. Results and Discussion

### 3.1. ZrO_2_ Layers

**Figure 1:**
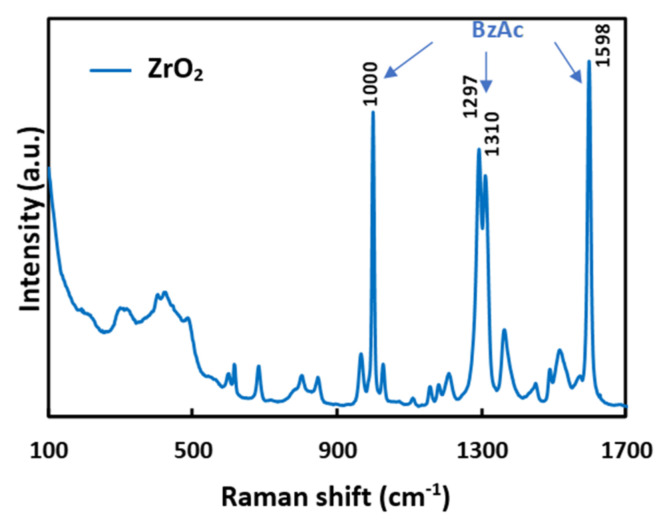
Raman spectrum of a ZrO_2_ xerogel thin film deposited on SiO_2_ substrate.

After spin-coating and drying at room temperature to evaporate solvents, the ZrO_2_ layer was analyzed using Raman spectroscopy. Figure 1 shows the Raman spectrum of the ZrO_2_ xerogel thin film deposited on silica substrate after annealing at 110 °C for 90 min. One can observe that the ZrO_2_ layer has an amorphous phase. Moreover, no peaks characteristic of a ZrO_2_ crystal phase were observed by XRD analysis (figure not shown), confirming the amorphous nature of the films. Indeed, the corresponding Raman spectrum (Figure 1) shows multiple peaks but no features of crystallized ZrO_2_. According to Oda et al. [37], the strong peaks at 1598 and 1000 cm^−1^ are assigned to 8b and 12 vibration modes of the phenyl group of BzAc and the peaks at 1297 and 1310 cm^−1^ are assigned to C=C=C symmetric vibrations in chelating rings [40]. The others small Raman peaks are supposed to be related to a BzAc or ZrO_2_/BzAc complex.

**Figure 2:**
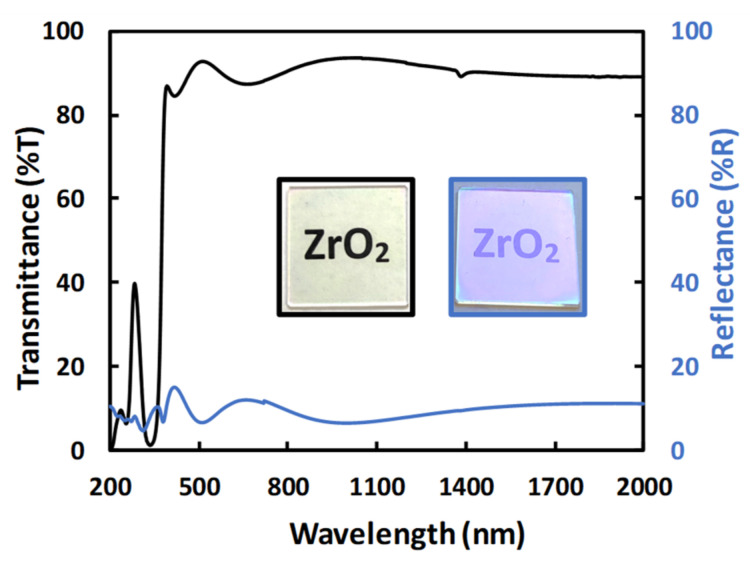
UV–Visible-NIR transmittance (in blue) and reflectance (in black) spectra of ZrO_2_ xerogel thin film deposited on SiO_2_ substrate. Inset: black and blue represent optical photographs of the ZrO_2_ layers in transmission and specular reflection, respectively.

Apart from Raman spectroscopy, the ZrO_2_ amorphous thin film was also analyzed with UV-Vis-NIR spectroscopy in the wavelength range of 200–2000 nm. Figure 2 shows the ZrO_2_ xerogel thin film spectra. The black and blue curves are, respectively, the transmission and reflection spectra of ZrO_2_ xerogel film deposited on silica substrate. The ZrO_2_ layers are transparent and slightly yellowish (Figure 2). Transmittance analyses carried out reveal a transparent oscillating region in the visible and near infrared with a maximum transmittance higher than 85% on the one hand, and a typical absorption of ZrO_2_ around 380 nm, where the transmittance decreases drastically, on the other hand [41]. The signal at 290 nm originates from the chelate ring (Zr(OC_4_H_9_)_4_ + BzAc) [41]. The reflectance curve of the ZrO_2_ film is in agreement with the results in transmission, with a reflectance lower than 15% and a slight purple color in reflection. The thickness of the layer was measured with a profilometer close to 300 nm.

### 3.2. Micro-Nanostructuration of ZrO_2_ Xerogel Films

#### 3.2.1. Optical Lithography

**Figure 3:**
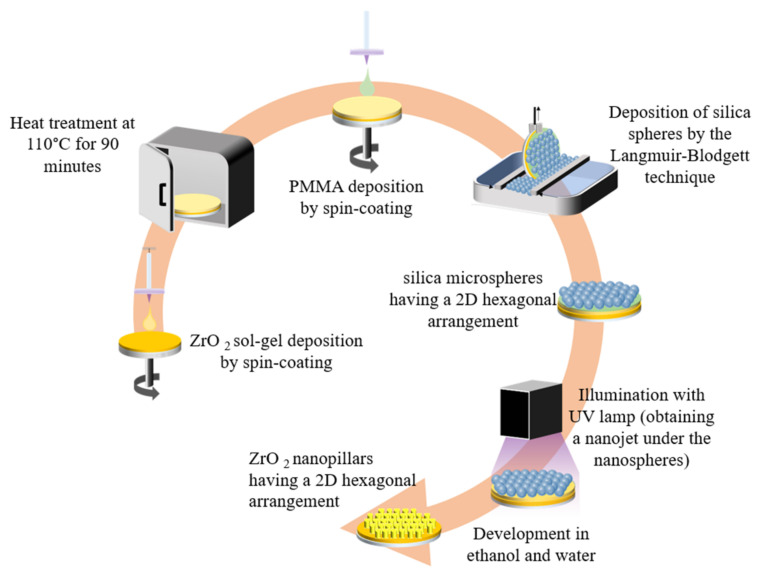
Illustration of the micro-nanostructuring process by colloidal lithography.

One way to obtain micro-nanostructured layers is to use the colloidal lithography technique in order to obtain amorphous ZrO_2_ nano-pillars, according to the process described in [35] (Figure 3).

**Figure 4:**
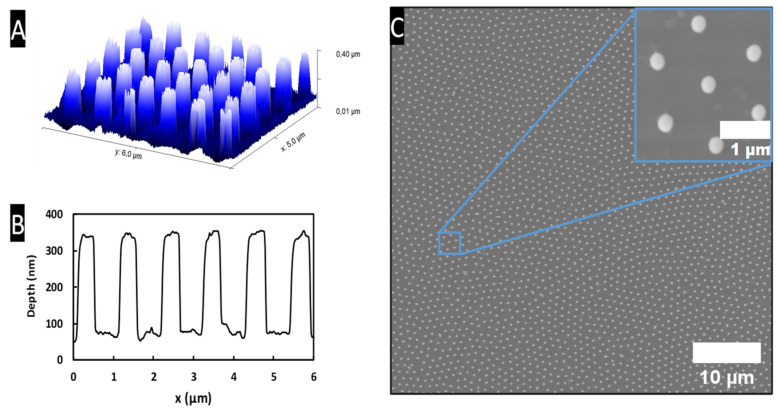
(**A**,**B**) Three-dimensional AFM image and profile of nano-structured ZrO_2_ thin film. (**C**) SEM top view image of the ZrO_2_ nano-pillars with an inset picture showing the hexagonal arrangement of the nano-pillars.

After homogeneous deposition of the silica microspheres following a 2D periodic hexagonal arrangement (in both *x* and *y* directions), the substrate was then illuminated with homogeneous UV light to create the nano-pillars (cylinder-shaped in the *z* direction) with an arrangement also following a 2D hexagonal pattern (Figure 4C). Each microsphere behaves like a micro-lens by focusing incident UV light and creates a photonic nano-jet that emerges underneath the microsphere in the ZrO_2_ layer [35]. After development and thermal stabilization of the ZrO_2_ layer at 110 °C for 1 h, a nano-structuring composed of nano-pillars is obtained within the hexagonal arrangement imposed by the nano-sphere deposition.

Figure 4C shows the nano-structured ZrO_2_ xerogel thin film after UV (λ = 365 nm) illumination and development of the xerogel layer, revealing periodically organized nano-pillars within a hexagonal arrangement. It is important to note that the 1 µm silica spheres used to make these films are not really mono-dispersed in size, which induces dislocations between small periodically well-arranged nano-sphere areas visible on the SEM image (Figure 4C). These nano-pillars appear to be fairly regular, with an average plot diameter calculated to 540 ± 30 nm with a periodicity of 1 µm corresponding to the microsphere diameter. From the 3D AFM image and profile of nano-structured ZrO_2_ thin film (Figure 4A,B), the nano-pillars have a cylindrical shape with a height of about 280 nm.

**Figure 5:**
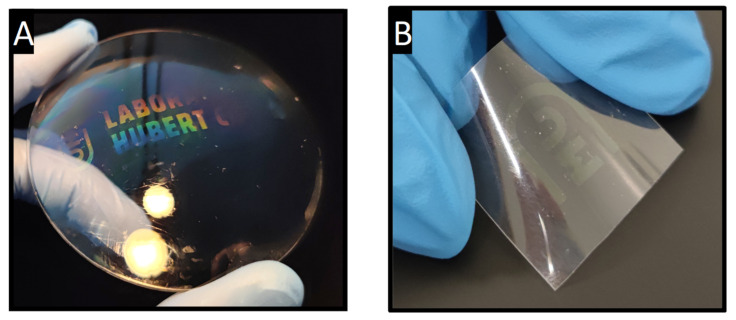
Examples of structuring on unconventional substrates: (**A**) ZrO_2_ multiscale pattern on a convex glass optical lens and (**B**) ZrO_2_ pattern on flexible plastic.

To extend the demonstration, the process was adapted to non-conventional substrates such as non-planar substrates. The versatile macro- and micro-structuring techniques described above have been applied to standard planar substrates. However, as shown in Figure 4, the process is also suitable for use on non-conventional substrates, such as lenses with varying degrees of curvature, or on flexible and bendable plastic sheets, while retaining their optical properties. Figure 5A shows a convex lens with a multiscale structured ZrO_2_ coating by combining macro- and micro-structuring (using colloidal lithography and UV illumination through a macroscopic mask before development and stabilization). Figure 5B presents a macroscopic ZrO_2_ pattern on a plastic sheet.

#### 3.2.2. Nano-Imprint Lithography

**Figure 6:**
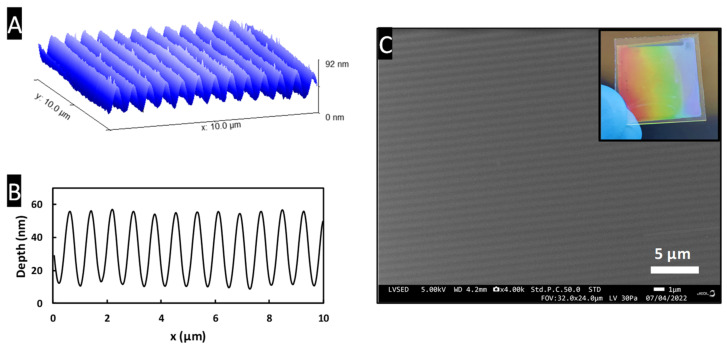
Example of structuring on planar glass substrates: ZrO_2_ xerogel sub-micronic diffraction grating. (**A**,**B**) AFM 3D image and profile of the nano-structured ZrO_2_ thin film. (**C**) SEM top view image of the ZrO_2_ diffraction grating with an inset picture showing the iridescence phenomenon of the structured ZrO_2_ layer.

Another method for structuring this ZrO_2_ xerogel is to use the direct UV nano-imprinting process to obtain, for example, amorphous ZrO_2_ diffraction grating. Figure 6 shows an example of structured ZrO_2_ xerogel thin layers obtained by nano-imprint. From PDMS stamps with a sinusoidal 1D grating of 800 nm period and 60 nm deep, it is possible to obtain replicas based on ZrO_2_ xerogel with similar characteristics to the ones of the stamp. AFM analysis illustrated in Figure 6A,B show that the ZrO_2_ replica has a period of around 800 nm with a depth close to 50 nm. Figure 6C shows the grating pattern obtained with the nano-imprint method demonstrating the good uniformity of the ZrO_2_ replica from both the microscopic SEM image and the good colored diffraction observed in the far field (in the −1st diffracted order direction) as shown in the inset.

**Figure 7:**
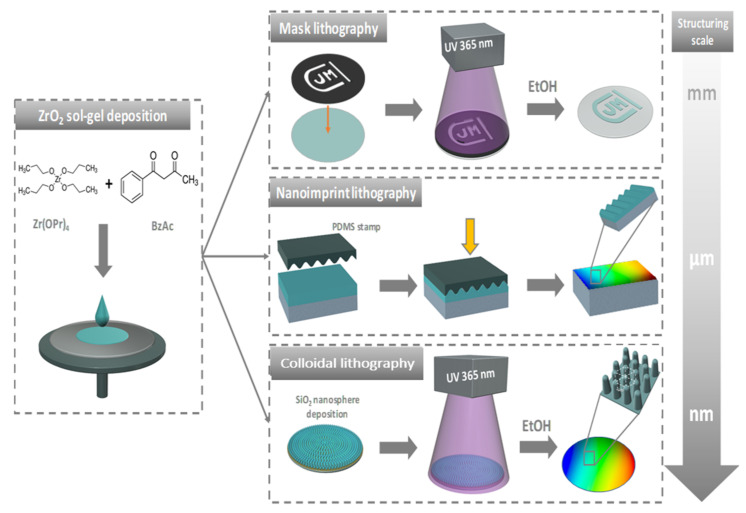
Illustration of the different methods of ZrO_2_ sol-gel micro-nanostructuring.

Figure 7 is an illustration summarizing the different possible methods of sol-gel micro-nanostructuring.

## 4. Conclusions

In conclusion we firstly demonstrated that the same ZrO_2_ sol-gel can be used both by optical and by nano-imprint lithography to realize complex patterns. This patterning by lithography is possible thanks to the presence of BzAc in the sol, which makes this xerogel layer photosensitive under UVA. The degradation of the BzAc makes the exposed areas insoluble in a solvent, thus inducing a contrast of solubility between the exposed and non-exposed areas. The xerogel then behaves as a negative photoresist, allowing its structuring at different scales by optical lithography or by nano-imprint lithography. We have also shown that after degradation of BzAc the ZrO_2_ films are amorphous.

Secondly, we have shown the possibility to micro-nanostructure ZrO_2_ xerogel films at different scales using optical mask or nanosphere lithography or a combination of both methods. Different patterns were realized on substrates varying in nature (plastic, glass, paper) and also in shape (planar and non-planar). Thirdly, patterns at different scales (millimeter and micrometer) were realized by nano-imprint lithography on planar substrates, opening the route to a cost-effective, fast, and direct micro-nanostructuring approach (without any etching processes) of functional coatings. This can allow an industrial development and an economic valorization of this sol-gel based process.

## Data Availability

Not applicable.
